# Neutrophil: lymphocyte ratio is positively associated with subclinical diabetic cardiomyopathy

**DOI:** 10.1186/s12902-020-00571-y

**Published:** 2020-06-30

**Authors:** Xiaoli Huang, Zihan Qin, Min Xu, Feifei Zhang, Xiaohong Jiang, Fei Hua, Lichan Tao

**Affiliations:** 1 Department of Endocrinology, The Third Affiliated Hospital of Soochow University, Changzhou City, 213003 China; 2 Department of Echocardiography, The Third Affiliated Hospital of Soochow University, Changzhou City, 213003 China; 3 Department of Nuclear Medicine, The Third Affiliated Hospital of Soochow University, Changzhou City, 213003 China; 4 Department of Cardiology, The Third Affiliated Hospital of Soochow University, Changzhou City, 213003 China

**Keywords:** NLR, Type 2 diabetes, Diabetic cardiomyopathy

## Abstract

**Background:**

Subclinical diabetic cardiomyopathy (DCM) occurs frequently in asymptomatic subjects with Type 2 diabetes mellitus (T2DM). The direct association between the immune system and DCM with effective biomarkers has been demonstrated in previous studies.

**Methods:**

Five hundred seven subjects with T2DM were recruited from April 2018 to October 2019 and divided into T2DM with cardiac dysfunction (DCM) group and T2DM without cardiac dysfunction (non-DCM) group. The relationship between the quartiles of Neutrophil: lymphocyte ratio (NLR) and subclinical DCM was evaluated by using adjusted logistic regression models.(covariates: age, sex, BMI, duration of diabetes, and hyperlipidemia).

**Results:**

Blood NLR was significantly upregulated in DCM group compared to non-DCM group (*P* = 0.05). Then the adjusted odds ratio (95% CI) of the highest NLR quartile was 14.32 (2.92–70.31) compared with the lowest quartile of NLR after multiple adjusted (*P* < 0.001). However, there was no significant relation between neutrophil and lymphocyte counts and the occurrence of DCM in T2DM patients.

**Conclusions:**

This study demonstrated that NLR was associated with the occurrence of subclinical DCM, suggesting that NLR may be a biomarker for predicting DCM with effectiveness and accuracy.

**Trial registration:**

Chinese Clinical Trial Registry (ChiCTR1900027080). Registered 30 October 2019. Retrospectively registered: www.medresman.org

## Background

Diabetic cardiomyopathy (DCM) is defined as a heart muscle-specific dysfunction arising independently of hypertension, coronary artery disease (CAD), or evidence of other structural cardiac diseases [1]. Recent studies showed that over 70% of diabetic patients will develop some form of cardiovascular disease during their lifetime [2]. Importantly, mounting evidence supports the occurrence of myocardial dysfunction even in asymptomatic T2DM (a condition also referred as subclinical DCM) [3–5], the occurrence of which is estimated to range from 50 to 70% [6, 7]. To design resultful prevention strategies, we should better understand early risk factors and predictors. It is consistent with what is strongly recommended in current clinical guidelines which emphasizes the significance of early diagnosis and effective intervention for diabetic patients who at risk.

Important pathological processes involved in DCM include cardiac hypertrophy, myocardial and interstitial fibrosis, oxidative stress, apoptosis, and microangiopathy [8, 9]. Activation of inflammatory mediators represents the major driver of all of the indicated pathological processes [8, 10–13]. Prior reports demonstrated increased levels of inflammatory cytokines in diabetic patients, such as c-reactive protein (CRP), interleukin (IL)-1β, IL-6, and tumor necrosis factor (TNF)-α [14, 15]. Furthermore, chronic low-grade inflammation may also lead to cardiometabolic disease by inducing insulin resistance, a major contributor to DCM development [16, 17]. Dysregulation of immune inflammatory factors, including elevated levels of acute-phase reactants [18], IL-6 [18], CRP [19], and cortisol [20], were also well associated with the cardiometabolic-risk profile. Chronic inflammation is therefore hypothesized to underlie the constellation of DCM risk factors [14]. However, current literature on the association between chronic inflammation and DCM in populations remains limited. Whether inflammation is involved in different stages of DCM is also unknown.

Neutrophil: lymphocyte ratio (NLR) has been recently identified as a novel biomarker of numerous inflammatory diseases [21–23]. Because of the convenience and affordability of NLR examination, it has been increasingly used in clinical testing and experimental research. Currently, the relationship between NLR and T2DM has been confirmed in two reports [24, 25]; Whereas, there have been few studies to examine the association between NLR and DCM, and it is uncertain whether NLR is related to different stages of DCM. Therefore, the present study is aimed to explore the possible associations between subclinical DCM and NLR, and to assess the power of NLR to predict for DCM.

## Methods

### Subject recruitment

This research was conducted as a cross-sectional, hospital-based study. A total of 532 subjects with T2DM were consecutively recruited from the Department of Endocrinology at the Third Affiliated Hospital of Soochow University (Changzhou, China) from April 2018 to October 2019, based on a priori protocol that was independent of the indications found with the echocardiographic examination. Of the 532 subjects with T2DM, 25 were excluded after gated-myocardial perfusion imaging (gated-MPI, including rest and stress) suggested the presence of occult CAD. All protocols were carried out in accordance with the principles outlined in the Declaration of Helsinki and the study was approved by the institutional review committee of the Third Affiliated Hospital of Soochow University. The ethics Committee of the Third Affiliated Hospital of Soochow University approved the study, which has been registered in the Chinese Clinical Trial Registry (ChiCTR1900027080).

The inclusion criteria for the subjects were: T2DM patients aged from 40 to 70 years, without regard to the duration of diabetes. The clinical treatment was composed of the oral use of anti-diabetic drugs and/or insulin injection. We excluded participants with one or more of the following characteristics: 1. A history of CAD, hypertension, valvular heart disease, atrial fibrillation, or any other cardiovascular disease. 2. A self-reported history of symptomatic macrovascular or microvascular complications of diabetes, including retinopathy, nephropathy, neuropathy, peripheral vascular disease, and stroke. 3. Pregnancy. 4. Other important comorbidities including infectious diseases, malignancy, thyroid dysfunction, hepatic and renal dysfunction, immune and rheumatic diseases, or significant psychiatric illness. All subjects signed informed consent prior to participating in the study.

### Trial design

On the same day of admission to the hospital (i.e., the basal day), patients with T2DM provided a complete medical history and were subjected to comprehensive physical and clinical examinations. On the following morning after admission and when the fasting time was greater than 8 h, we collected the patient’s peripheral blood samples. All patients underwent electrocardiography (ECG), echocardiography, and gated-myocardial perfusion imaging (gated-MPI, including rest and stress). Eligible subjects with evidence of left ventricular diastolic dysfunction (LVDD) and no apparent occult CAD were defined as having subclinical DCM. We classified LVDD according to recommendations of the American Society of Echocardiography and the European Association of Cardiovascular Imaging [26]. All patients underwent rest and stress gated-MPI in the presence of a cardiologist so that we could exclude CAD. The criteria for defining ischemia based on the gated-MPI results were defined according to the 2016 American Society of Nuclear Cardiology imaging guidelines for single-photon emission computed tomography-based nuclear cardiology procedures [27]. All patients underwent rigorous analysis of clinical variables, metabolic measurements, echocardiography, pre- and post-exercise myocardial function during the baseline-screening phase.

### Demographic, clinical, and metabolic data

Demographic data were analyzed regarding the patients’ sex, age, basic anthropometry (including body–mass index [BMI] and waist-to-hip circumference ratio), duration of diabetes and concomitant hypoglycemic drug use. The clinical data obtained included the levels of B-type natriuretic peptide (BNP, a marker of heart failure) and myocardial enzymes. Biochemical analysis was performed to determine the levels of alanine aminotransferase (AST), glutamic oxaloacetic transaminase (ALT), creatinine, urea nitrogen (BUN), and creatinine (Cr). Metabolic measurements included fasting-blood glucose (FBG), postprandial-blood glucose (PBG), HbA1c, and lipid profiles. Briefly, HbA1c was measured with boronate affinity high-performance liquid chromatography. HbA1c values were corrected on the basis of the correlation coefficient that was derived from a validation experiment that used sample data that were measured on both analysers. Serum Cr, BUN, myocardial enzymes, BNP, ALT, AST, and lipid profiles including triglyceride, total cholesterol, HDL-cholesterol, LDL-cholesterol were measured on a Modular P800 anayser (Roche Diagnostics, Mannheim, Germany). The clinical and metabolic data were measured after fasting for at least 8 h and before administering hypoglycemic agents.

### Echocardiography and data analysis

The following echocardiography parameters were determined: the left atrial diameter (LAD), left ventricular end-diastolic diameter (LVEDD), left ventricular end-systolic diameter (LVESD), interventricular septal diameter (IVSD), left ventricular ejection fraction (LVEF), left atrial mass (LA mass), LA mass index, peak early diastolic trans-mitral flow velocity (E), peak late diastolic trans-mitral flow velocity (A), and peak early diastolic mitral annular velocity (e′). The E/A ratio, E/e′ ratio, and LA volume indexes were calculated to evaluate the diastolic function.

### Gated-MPI

Gated stress–rest ^99m^TC-MIBI MPI was performed for each subject based on a 2-day standard imaging protocol [27]. Regardless of the stress or rest examinations, we collected images after 99mTC-MIBI injection at a dose of 740–925 MBq for 60–90 min. According to the modified Bruce protocol, the exercise stress test was performed on a bicycle, and the pharmacological stress test was performed with injection of adenosine intravenously (140 μg•kg– 1•min– 1) within 6 min. Based on standard guidelines, two nuclear physicians with extensive experience interpreted images of the short-axis, horizontal-axis, and vertical long-axis all alone according to qualitative visual interpretation. Neither physician knows the clinical data of each patient. When there was a disagreement, a third expert was recruited to resolve the discrepancies. Myocardial ischemia was defined as reversible sparsity or defects on two different fault planes and two consecutive layers of stress–rest tomography at the same location, whereas myocardial infarction was defined as irreversible sparsity or defects on two different fault planes and two consecutive layers of stress–rest tomography at the same location.

### NLR analysis

We determined neutrophil and leukocyte using an automated hematology analyzer. Afterwards, the neutrophil/lymphocyte ratio (NLR) was obtained by dividing the neutrophil by the lymphocyte count. The participants were divided into four groups based on their quartile to inquiry how NLR, neutrophil and lymphocyte counts correlate with the prevalence and occurrence of DCM.

### Statistical analysis

We used SPSS software (version 23.0) to analyze all clinical data. For continuous, normally distributed variables, the results are presented as the mean ± SD. For categorical variables, the results are presented as percentages. To perform further analysis, we used the occurrence of DCM as a dependent variable, and the quartiles of NLR, neutrophil, and lymphocyte counts as independent variables. For baseline characteristics of patients, the differences among quartiles of NLR were analyzed using ANOVA followed by Bonferroni’s post-hoc test for continuous variables and logistic regression analysis for proportional variables. The relationship between quartiles of NLR and the incidence of DCM was examined with multiple logistic regression analysis after the adjustment of covariates, which consisted of age, sex, BMI, duration of diabetes and hyperlipidemia. Odds ratio (OR) (95% CI) were calculated. We evaluated the sensitivity and specificity of the NLR to predict the diagnosis of DCM by generating a receiver-operator characteristic curve (ROC). A *P* value < 0.05 was deemed to be statistically significant.

## Results

### Patients’ characteristics

In the cross-sectional study, 532 subjects were screened from April 2018 to October 2019. Of the 532 subjects with T2DM, 25 were excluded after gated-MPI suggested the presence of occult CAD. We divided the 507 remaining subjects into T2DM with cardiac dysfunction (DCM) group (*n* = 465) and T2DM without cardiac dysfunction (non-DCM) group (*n* = 45). For the clinical characteristics of subjects, statistically significant differences were detected in terms of sex, age, BMI, duration of diabetes, FBG, 2-h PBG, and lipid profiles including TC, HDL and LDL (all *P* value < 0.05) (Supplemental Table 1) between the two groups. Echocardiographic features of subjects with or without cardiac dysfunction were presented in Supplemental Table 2 and significant difference were observed in cardiac structure (IVSD), EF%, and cardiac diastolic function including A velocity, E/A ratio, e’ velocity, and E/e’ ratio (all *P* value < 0.05).

Furthermore, NLR was upregulated in T2DM subjects with cardiac dysfunction (Supplemental Table 1). Then we assessed clinical characteristics across NLR quartiles in Table 1. Subjects in the upper three quartiles of NLR had a trend to have longer duration of diabetes, to have higher 2-h FBG, HbA1c%, total cholesterol (TC) and triglyceride (TG) (all *P* value < 0.05) than subjects in the lowest quartile.. Different from above results, there was no important differences among the subjects in different NLR quartiles.

**Table 1:** Participant characteristics by quartile of neutrophil: lymphocyte ratio (NLR) (*n* = 507)

Participant characteristics	Quartiles of NLR (range)				
	Level 1 (< 1.35) *n* = 124	Level 2 (≥1.35 and < 1.77) *n* = 129	Level 3 (≥1.77 and < 2.38) n = 127	Level 4 (≥2.38) n = 127	*P* value
Sex (male, %)	80 (64.52%)	79 (61.24%)	84 (66.14%)	70 (55.12%)	0.894
Age (years)	54.84 ± 8.16	54.84 ± 8.16	54.84 ± 8.16	54.84 ± 8.16	0.083
BMI (kg/m^2^)	23.29 ± 3.43	23.64 ± 3.29	23.52 ± 3.87	23.64 ± 3.64	0.935
WHR	1.01 ± 0.91	0.93 ± 0.70	0.94 ± 0.06	0.93 ± 0.06	0.430
Duration of diabetes (years)	5.53 ± 4.68	6.47 ± 6.24	7.24 ± 5.35	7.79 ± 5.29	0.002*
FBG (mmol/L)	9.99 ± 3.03	9.58 ± 3.03	9.27 ± 2.90	9.86 ± 3.26	0.246
2-h PBG (mmol/L)	13.80 ± 4.40	14.78 ± 5.45	13.57 ± 4.94	15.12 ± 4.96	0.023*
HbA1c (%)	9.41 ± 1.75	9.62 ± 2.23	9.80 ± 2.31	10.28 ± 2.51	0.026*
ALT (U/L)	23.43 ± 14.13	24.98 ± 26.39	21.97 ± 17.08	20.69 ± 13.64	0.268
AST (U/L)	23.35 ± 11.87	24.09 ± 25.71	21.51 ± 12.70	20.27 ± 11.75	0.250
BUN (mmol/L)	5.21 ± 1.30	5.25 ± 1.29	5.39 ± 1.46	5.32 ± 1.53	0.653
Cr (mmol/L)	69.33 ± 14.99	67.66 ± 13.07	69.93 ± 12.48	70.53 ± 13.45	0.506
BNP (ng/L)	33.19 ± 22.25	33.43 ± 27.71	35.52 ± 27.51	39.08 ± 25.71	0.102
CTNI (ng/mL)	0.0017 ± 0.002	0.0025 ± 0.006	0.0025 ± 0.004	0.0016 ± 0.002	0.637
CK (U/L)	74.75 ± 42.10	77.00 ± 43.86	65.44 ± 20.29	72.80 ± 35.44	0.540
CK-MB (U/L)	1.47 ± 0.99	1.48 ± 0.84	1.66 ± 2.33	1.41 ± 0.79	0.488
Myoglobin (ng/mL)	20.16 ± 11.30	19.45 ± 8.68	22.10 ± 11.47	19.47 ± 7.92	0.235
TC (mmol/L)	4.43 ± 1.09	4.63 ± 1.36	4.74 ± 0.94	4.89 ± 2.51	0.018*
TG (mmol/L)	1.84 ± 1.37	1.91 ± 1.37	2.38 ± 2.56	2.85 ± 1.90	0.039*
HDL (mmol/L)	1.10 ± 0.35	1.09 ± 0.36	1.03 ± 0.29	1.09 ± 0.32	0.190
LDL (mmol/L)	2.55 ± 0.80	2.72 ± 0.81	2.78 ± 0.62	2.82 ± 0.77	0.420
Hypoglycemic drug use (%)					
Sulfonylurea	24 (19.35%)	23 (17.83%)	24 (18.90%)	20 (15.75%)	0.741
Metformin	100 (80.65%)	105 (81.40%)	103 (81.10%)	103 (81.10%)	0.500
α-glucosidase inhibitor	104 (83.87%)	110 (85.27%)	112 (88.19%)	109 (85.83%)	0.764
GLP-1	18 (14.52%)	17 (13.18%)	19 (14.96%)	19 (14.96%)	0.636
DPP4 inhibitor	11 (8.87%)	12 (9.30%)	13 (10.24%)	13 (10.24%)	0.473
SGLT2 inhibitor	11 (8.87%)	13 (10.08%)	11 (8.66%)	12 (9.45%)	0.349
Insulin	88 (70.97%)	93 (72.09%)	91 (71.65%)	90 (70.87%)	0.523

The data are summarized as the mean ± SD for continuous variables or as a numerical proportion for categorical variables. *BMI* body mass index, *WHR* waist-to-hip ratio, *FBG* fasting blood glucose; *2-h PBG* 2-h postprandial blood glucose, *HbA1c* hemoglobin A1c, *ALT* alanine aminotransferase, *AST* aspartate aminotransferase, *BNU* urea nitrogen, *Cr* creatinine, *BNP* type B natriuretic peptide, *CTNI* cardiac troponin I, *CK* creatine kinase, *TC* total cholesterol, *TG* triglyceride, *HDL* high density lipoprotein, *LDL* low density lipoprotein

Table 2 presented the cardiac structure and LV function of patients with T2DM across NLR quartiles are presented. In comparison with subjects in the lowest quartile of NLR, significant differences in IVSD were observed in the upper three quartiles (*P* = 0.002). In terms of LV function, subjects in the upper three quartiles tended to have E/A ratio and e’ velocity, but higher A velocity and E/e’ ratio (all *P* value < 0.001). However, E velocity showed no statistical significance across NLR quartiles.

**Table 2:** Echocardiographic features of participants by quartile of neutrophil: lymphocyte ratio (NLR) (*n* = 507)

Participant characteristics	Quartiles of NLR (range)				
	Level 1 (< 1.35) *n* = 124	Level 2 (≥1.35 and < 1.77) *n* = 129	Level 3 (≥1.77 and < 2.38) *n* = 127	Level 4 (≥2.38) n = 127	*P* value
LA diameter	(1)	33.88 ± 3.35	33.81 ± 3.09	33.76 ± 3.25	0.918
LVEDD (mm)	45.63 ± 2.07	45.58 ± 2.82	45.99 ± 2.93	46.35 ± 3.48	0.143
LVESD (mm)	29.71 ± 1.45	29.64 ± 1.55	29.96 ± 1.48	30.41 ± 2.37	0.002*
IVSD (mm)	8.30 ± 0.57	8.10 ± 0.55	8.38 ± 0.64	8.74 ± 1.18	< 0.001*
EF (%)	64.58 ± 1.79	64.23 ± 1.55	64.43 ± 1.64	64.27 ± 2.24	0.418
E velocity (cm/s)	76.24 ± 14.66	75.43 ± 11.19	75.42 ± 13.99	76.30 ± 15.60	0.934
A velocity (cm/s)	66.31 ± 13.77	68.02 ± 15.17	75.92 ± 16.69	87.66 ± 12.91	< 0.001*
E/A ratio	1.16 ± 0.14	1.13 ± 0.17	1.03 ± 0.23	0.88 ± 0.22	< 0.001*
e’ velocity (cm/s)	9.92 ± 1.66	9.69 ± 1.78	9.20 ± 1.96	8.32 ± 1.79	< 0.001*
E/e’ ratio	7.71 ± 0.99	7.86 ± 1.10	8.39 ± 1.56	9.29 ± 1.39	< 0.001*

The data are summarized as the mean ± SD for continuous variables or as a numerical proportion for categorical variables. *LA* left atrial, *LV* left ventricular, *LVEDD* LV diameter in end diastolic, *LVESD* LV diameter in end systolic, *IVSD* interventricular septal diameter, *LVEF* LV ejection fraction, *E* the peak early diastolic trans-mitral flow velocity, *A* the peak late diastolic trans-mitral flow velocity, *e’* the peak early diastolic mitral annular velocity

### Association of NLR with risk of subclinical DCM in patients with T2DM

**Fig. 1:**
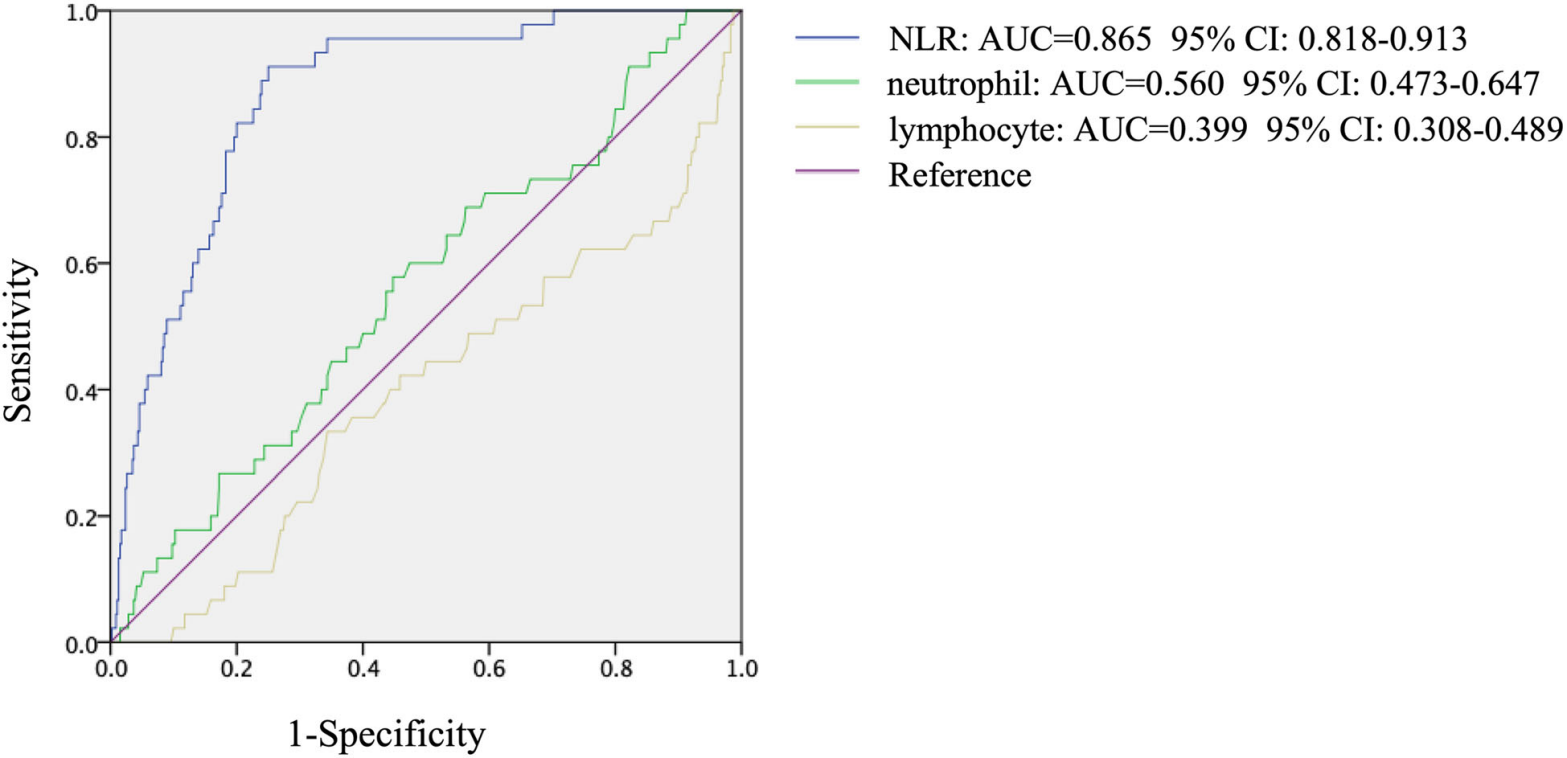
Receiver-operator characteristic (ROC) curve analysis of NLR, neutrophil and lymphocyte in type 2 diabetes with cardiac dysfunction versus type 2 diabetes without cardiac dysfunction

The rough and adjusted relations between quartiles of NLR and DCM were presented in Table 3. In the final multivariate models, which adjusted for age, sex, BMI, duration of diabetes and serum lipid, the ORs (95% CI) for DCM across NLR quartiles were 1.00 (reference), 8.02 (1.53, 41.96), 12.55 (2.52, 62.35), and 14.32 (2.92, 70.31) (*P* = 0.001). Furthermore, we performed ROC analysis following AUC to assess the power of NLR (AUC = 0.865, 95% CI: 0.818, 0.913), neutrophil (AUC = 0.560, 95% CI: 0.473, 0.647) and lymphocyte (AUC = 0.399, 95% CI: 0.308, 0.489) to discriminate T2DM patients with or without cardiac dysfunction (Fig. 1 and Supplemental Table 3). NLR, rather than neutrophil or lymphocyte, was identified as a potential predictor of DCM.

**Table 3:** Adjusted relationships of quartiles of neutrophil: lymphocyte ratio (NLR) to diabetic cardiomyopathy (DCM) (*n* = 507)

Adjusted relationships	Quartiles of NLR (range)				
	Level 1 (< 1.35) *n* = 124	Level 2 (≥1.35 and < 1.77) *n* = 129	Level 3 (≥1.77 and < 2.38) *n* = 127	Level 4 (≥2.38) n = 127	*P* value
Number of DCM	2	9	16	18	–
Crude	1.00 (Ref)	4.57 (0.96–21.59)	8.79 (1.98–39.07)	10.01 (2.28–44.47)	< 0.001*
Model 1	1.00 (Ref)	5.01 (1.04–24.09)	10.29 (2.26–46.77)	13.43 (2.96–60.95)	< 0.001*
Model 2	1.00 (Ref)	5.47 (1.11–27.02)	11.59 (2.48–54.25)	16.16 (3.46–75.36)	< 0.001*
Model 3	1.00 (Ref)	8.02 (1.53–41.96)	12.55 (2.52–62.35)	14.32 (2.92–70.31)	< 0.001*

Model 1: age-, sex-, and BMI- adjusted;

Model 2: age-, sex-, BMI-, and duration of diabetes- adjusted;

Model 3: multiple adjusted, including age-, sex-, BMI-, duration of diabetes-, and lipid profile

**Fig. 2:**
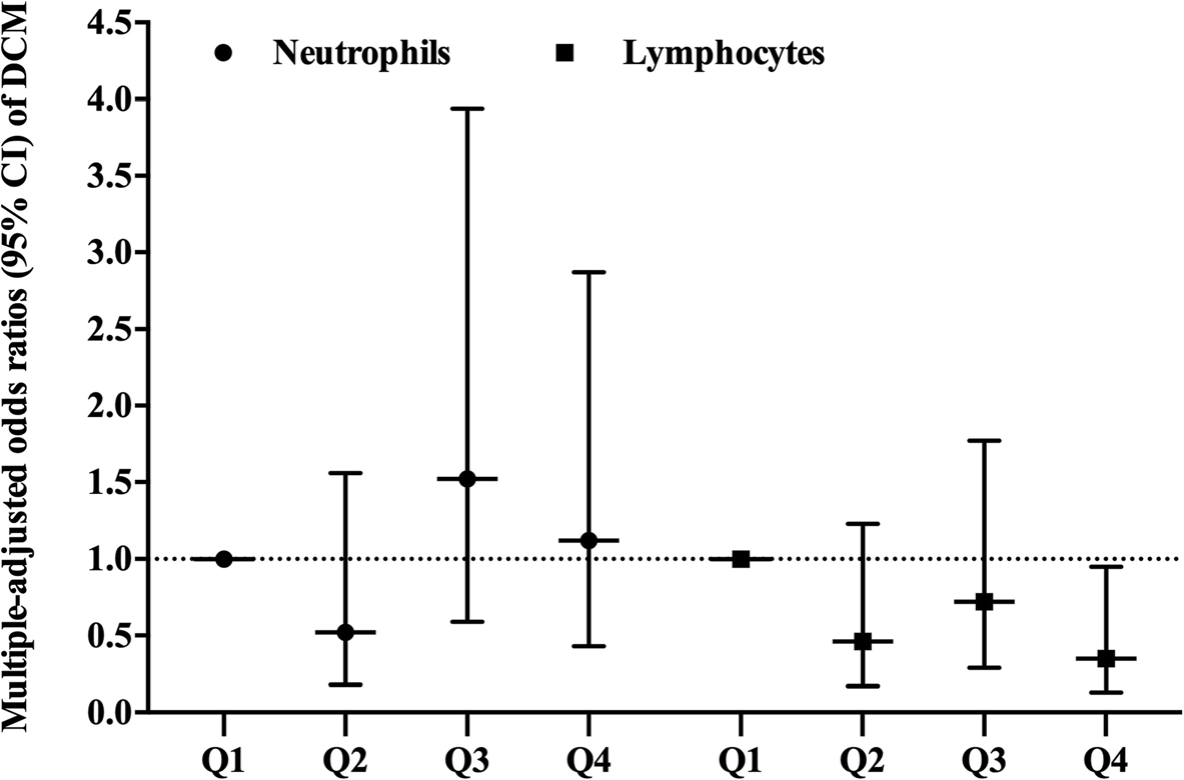
Adjusted Odds Ratio (OR) (95% CI) between the quartiles of neutrophil and lymphocyte counts and the occurrence of subclinical DCM. Adjusted for age, sex, BMI, duration of diabetes and hyperlipidemia (total cholesterol ≥5.17 mmol/l, triglyceride ≥1.7 mmol/l, LDL ≥ 3.37 mmol/l)

In addition, neutrophil and lymphocyte counts were divided into the following four categories based on participants’ quartiles (range × 1000 cells/mm^3^): neutrophil: < 2.68, 2.68–3.34, 3.34–4.18, and > 4.18. Lymphocyte: < 1.54, 1.54–1.90, 1.90–2.30, and > 2.30. After adjusting for multiple variants, the ORs (95% CI) of DCM for increasing quartiles of neutrophil counts were: 1.00, 0.52 (0.18, 1.56), 1.52 (0.59, 3.94), 1.12 (0.43, 2.87). For lymphocyte counts: 1.00, 0.46 (0.17, 1.23), 0.72 (0.29, 1.77), 0.35 (0.13, 0.95) (Fig. 2).

## Discussion

In this study, there is a positive relation between NLR, rather than neutrophil and lymphocyte counts, and the prevalence of DCM. This was the first study to relate the risk of subclinical DCM to NLR, which is an inflammation-related indicators applied widely to the medical field.

Recently, NLR has been identified as a useful biomarker in numerous chronic inflammatory diseases including diabetes, which reflects both an increase in the neutrophil counts and a reduction in the lymphocyte counts. For example, a high baseline NLR may serve as a useful indicator for short survival duration in patients with amyotrophic lateral sclerosis (ALS) [28]. Pretreatment NLR and albumin-to-gamma-glutamyl transferase ratio (AGR) predict the diagnosis of prognosis of Grade III oligodendroglial gliomas [22]. In a large-scale cross-sectional cohort study, NLR was significantly increased in diabetic patients and might serve as a biomarker with efficiency and accuracy for predicting T2DM [24]. Moreover, the role of NLR as a medical biomarker for diabetic complications has been confirmed by data from a few of studies. In research conducted by Sukhija et al., the NLR was used to identify individuals who have a risk of sensorineural hearing loss (caused by diabetic vascular complications via inflammation) [29]. In type 2 diabetic nephropathy (T2DN), it’s claimed that NLR was a marker for prognosticating T2DN outcomes [30], including microvascular complications [31] and impaired renal function [32]. In this study, it’s the NLR, rather than neutrophil and lymphocyte counts, that was associated with the incidence of subclinical DCM among T2DM patients independently and significantly. Determining the NLR is both simple and affordable; thus, it could predict DCM innovatively and effectively.

Previous findings demonstrated that the first manifestation of myocardial involvement in diabetes was preclinical LV diastolic dysfunction (LVDD) [33, 34]. Although myocardial changes can be detected by echocardiography, several cardiac abnormalities can occur even before the onset of structural and hemodynamic dysfunction in T2DM patients without LVDD. A much more focused approach is therefore needed for T2DM patients, involving regularly assessing systemic abnormalities and cardiovascular risk in this population. One such approach would be to employ circulating biomarkers to track diabetes progression and for medication guidance. Increasing evidence suggest that inflammation is involved in the pathophysiology diabetes and heart failure, and inflammation has emerged as a central theme in the pathology of systolic heart disease in recent decades [35, 36]. However, few studies have demonstrated the participation of inflammatory factors in the development of diastolic dysfunction [37], especially in diabetes-induced LVDD. In the present study, NLR positively correlated with impaired LVDD, which provide an independent association with subclinical DCM, and would provide effective prevention strategies, as strongly recommended in clinical guidelines.

### Limitations

The main limitation of this study was a deficiency of echocardiographic data and NLR value for patients with DCM at different stages. Echocardiography is the most common and widely used examination to evaluate cardiac structure and function, for asymptomatic DCM patients, it is necessary to have a regular echocardiography to evaluate diastolic and systolic function. However, owing to its relatively high price and a certain error measured by different doctors hamper its largescale application for early detection of asymptomatic DCM patients. NLR is both accessible and affordable, therefore, how to combine echocardiography data and NLR value to early predict DCM is an important clinical task and needs further exploration. Second, although NLR was identified as a significant and independent biomarker for diagnosing DCM, We still need to clarify the exact mechanisms of the ralationship between systemic inflammation and the prevalent conditions. Third, this study was limited by a single center and the findings need to be confirmed in larger and multi-center cohort studies in the future.

## Conclusions

To the best of our knowledge, our study provided the first evidence of a relationship between NLR and abnormal diastolic performance in T2DM patients without clinical symptoms. NLR was identified as an independent and early biomarker for the occurrence of DCM] in blood samples of subjects with T2DM. At present, it is not clear which mechanism(s) can explain the association between diastolic abnormalities and NLR. Therefore, the relationship between LVDD and activation of the immunoinflammatory system should be studied more thoroughly in the future, both on experimental and clinical grounds.

## Supplementary information

**Additional file 1 : Supplemental Table 1**. Participant characteristics of T2DM with or without cardiac dysfunction. **Supplemental Table 2**. Echocardiographic features of participants with or without cardiac dysfunction. **Supplemental Table 3**. ROC analysis for continuous predictor.

## Data Availability

The datasets are available from the corresponding author on reasonable request.

## Abbreviations

DCM: Diabetic cardiomyopathy
T2DM: Type 2 diabetes mellitus
NLR: Neutrophil: lymphocyte ratio
CAD: Coronary artery disease
TNF-α: Tumor necrosis factor α
Gated-MPI: Gated-myocardial perfusion imaging
ECG: Electrocardiography
LVDD: Left ventricular diastolic dysfunction
BMI: Body–mass index
BNP: B-type natriuretic peptide
AST: Alanine aminotransferase
ALT: Glutamic oxaloacetic transaminase
BUN: Urea nitrogen
Cr: Creatinine
FBG: Fasting-blood glucose
PBG: Postprandial-blood glucose
LAD: Left atrial diameter
LVEDD: Left ventricular end-diastolic diameter
LVESD: Left ventricular end-systolic diameter
IVSD: Interventricular septal diameter
LVEF: Left ventricular ejection fraction
LA mass: Left atrial mass
E: Peak early diastolic trans-mitral flow velocity
A: Peak late diastolic trans-mitral flow velocity
e’: Peak early diastolic mitral annular velocity
OR: Odds ratio
ROC: Receiver–operator characteristic
ALS: Amyotrophic lateral sclerosis
AGR: Albumin-to-gamma-glutamyl transferase ratio
T2DN: Type 2 diabetic nephropathy

## References

[1] Paolillo S, Marsico F, Prastaro M, Renga F, Esposito L, De Martino F (2019). Diabetic cardiomyopathy: definition, diagnosis, and therapeutic implications. Heart Fail Clin.

[2] Amsterdam EA, Wenger NK, Brindis RG, Casey DE, Ganiats TG, Holmes DR (2014). 2014 AHA/ACC guideline for the management of patients with non-ST-elevation acute coronary syndromes: executive summary: a report of the American College of Cardiology/American Heart Association task force on practice guidelines. Circulation..

[3] Fang ZY, Schull-Meade R, Downey M, Prins J, Marwick TH (2005). Determinants of subclinical diabetic heart disease. Diabetologia..

[4] Jellis CL, Jenkins C, Leano R, Martin JH, Marwick TH (2010). Reduced end-systolic pressure-volume ratio response to exercise: a marker of subclinical myocardial disease in type 2 diabetes. Circ Cardiovasc Imaging.

[5] Giorda CB, Cioffi G, de Simone G, Di Lenarda A, Faggiano P, Latini R (2011). Predictors of early-stage left ventricular dysfunction in type 2 diabetes: results of DYDA study. Eur J Cardiovasc Prev Rehabil.

[6] Faden G, Faganello G, De Feo S, Berlinghieri N, Tarantini L, Di Lenarda A (2013). The increasing detection of asymptomatic left ventricular dysfunction in patients with type 2 diabetes mellitus without overt cardiac disease: data from the SHORTWAVE study. Diabetes Res Clin Pract.

[7] Cioffi G, Giorda CB, Chinali M, Di Lenarda A, Faggiano P, Lucci D (2012). Analysis of midwall shortening reveals high prevalence of left ventricular myocardial dysfunction in patients with diabetes mellitus: the DYDA study. Eur J Prev Cardiol.

[8] Low A, Mak E, Rowe JB, Markus HS, O'Brien JT (2019). Inflammation and cerebral small vessel disease: a systematic review. Ageing Res Rev.

[9] Zhang W, Xu W, Feng Y, Zhou X (2019). Non-coding RNA involvement in the pathogenesis of diabetic cardiomyopathy. J Cell Mol Med.

[10] Nikolajevic Starcevic J, Janic M, Sabovic M. Molecular Mechanisms Responsible for Diastolic Dysfunction in Diabetes Mellitus Patients. Int J Mol Sci. 2019;20(5):1197. 10.3390/ijms20051197 PMC6429211 30857271

[11] Humeres C, Frangogiannis NG (2019). Fibroblasts in the infarcted, remodeling, and failing heart. JACC Basic Transl Sci.

[12] Davidovich P, Kearney CJ, Martin SJ (2014). Inflammatory outcomes of apoptosis, necrosis and necroptosis. Biol Chem.

[13] Hussain T, Tan B, Yin Y, Blachier F, Tossou MC, Rahu N (2016). Oxidative stress and inflammation: what polyphenols can do for us?. Oxidative Med Cell Longev.

[14] de Rooij SR, Nijpels G, Nilsson PM, Nolan JJ, Gabriel R, Bobbioni-Harsch E (2009). Low-grade chronic inflammation in the relationship between insulin sensitivity and cardiovascular disease (RISC) population: associations with insulin resistance and cardiometabolic risk profile. Diabetes Care.

[15] Garcia C, Feve B, Ferre P, Halimi S, Baizri H, Bordier L (2010). Diabetes and inflammation: fundamental aspects and clinical implications. Diabetes Metab.

[16] Jia G, Whaley-Connell A, Sowers JR (2018). Diabetic cardiomyopathy: a hyperglycaemia- and insulin-resistance-induced heart disease. Diabetologia..

[17] Jia G, DeMarco VG, Sowers JR (2016). Insulin resistance and hyperinsulinaemia in diabetic cardiomyopathy. Nat Rev Endocrinol.

[18] Pickup JC, Mattock MB, Chusney GD, Burt D (1997). NIDDM as a disease of the innate immune system: association of acute-phase reactants and interleukin-6 with metabolic syndrome X. Diabetologia..

[19] Suhett LG, Hermsdorff HHM, Rocha NP, Silva MA, Filgueiras MS, Milagres LC (2019). Increased C-reactive protein in Brazilian children: association with Cardiometabolic risk and metabolic syndrome components (PASE study). Cardiol Res Pract.

[20] Park MH, Park SI, Kim JH, Yu J, Lee EH, Seo SR (2019). The acute effects of hydrocortisone on cardiac electrocardiography, action potentials, intracellular calcium, and contraction: the role of protein kinase C. Mol Cell Endocrinol.

[21] Paliogiannis P, Fois AG, Sotgia S, Mangoni AA, Zinellu E, Pirina P, et al. Neutrophil to lymphocyte ratio and clinical outcomes in COPD: recent evidence and future perspectives. Eur Respir Rev. 2018;27(147):170113. 10.1183/16000617.0113-2017 PMC9488932 29436405

[22] He ZQ, Duan H, Lin FH, Zhang J, Chen YS, Zhang GH (2019). Pretreatment neutrophil-to-lymphocyte ratio plus albumin-to-gamma-glutamyl transferase ratio predict the diagnosis of grade III glioma. Ann Transl Med.

[23] Kang JW, Kim MG, Kim SS, Im HI, Dong SH, Kim SH, et al. Neutrophil-lymphocyte ratio as a valuable prognostic marker in idiopathic sudden sensorineural hearing loss. Acta Otolaryngol. 2019:1–7. 10.1080/00016489.2019.1705998. 10.1080/00016489.2019.1705998 31876220

[24] Guo X, Zhang S, Zhang Q, Liu L, Wu H, Du H (2015). Neutrophil:lymphocyte ratio is positively related to type 2 diabetes in a large-scale adult population: a Tianjin chronic Low-grade systemic inflammation and health cohort study. Eur J Endocrinol.

[25] Mertoglu C, Gunay M (2017). Neutrophil-lymphocyte ratio and platelet-lymphocyte ratio as useful predictive markers of prediabetes and diabetes mellitus. Diab Metab Syndr.

[26] Nagueh SF, Smiseth OA, Appleton CP, Byrd BF, Dokainish H, Edvardsen T (2016). Recommendations for the evaluation of left ventricular diastolic function by echocardiography: an update from the American Society of Echocardiography and the European Association of Cardiovascular Imaging. J Am Soc Echocardiogr.

[27] Henzlova MJ, Duvall WL, Einstein AJ, Travin MI, Verberne HJ (2016). ASNC imaging guidelines for SPECT nuclear cardiology procedures: stress, protocols, and tracers. J Nuci Cardiol.

[28] Choi SJ, Hong YH, Kim SM, Shin JY, Suh YJ, Sung JJ (2020). High neutrophil-to-lymphocyte ratio predicts short survival duration in amyotrophic lateral sclerosis. Sci Rep.

[29] Sukhija R, Aronow WS, Sorbera C, Peterson SJ, Frishman WH, Cohen M (2007). Mortality, left ventricular ejection fraction, and prevalence of new left ventricular wall motion abnormality at long-term follow-up in patients with implantable cardioverter defibrillators treated with biventricular pacing versus right ventricular pacing. Am J Ther.

[30] Ulu SM, Dogan M, Ahsen A, Altug A, Demir K, Acarturk G (2013). Neutrophil-to-lymphocyte ratio as a quick and reliable predictive marker to diagnose the severity of diabetic retinopathy. Diabetes Technol Ther.

[31] Ozturk ZA, Kuyumcu ME, Yesil Y, Savas E, Yildiz H, Kepekci Y (2013). Is there a link between neutrophil-lymphocyte ratio and microvascular complications in geriatric diabetic patients?. J Endocrinol Investig.

[32] Azab B, Daoud J, Naeem FB, Nasr R, Ross J, Ghimire P (2012). Neutrophil-to-lymphocyte ratio as a predictor of worsening renal function in diabetic patients (3-year follow-up study). Renal fail.

[33] Kozakova M, Morizzo C, Fraser AG, Palombo C (2017). Impact of glycemic control on aortic stiffness, left ventricular mass and diastolic longitudinal function in type 2 diabetes mellitus. Cardiovas Diabetol..

[34] Rawal S, Nagesh PT, Coffey S, Van Hout I, Galvin IF, Bunton RW (2019). Early dysregulation of cardiac-specific microRNA-208a is linked to maladaptive cardiac remodelling in diabetic myocardium. Cardiovasc Diabetol.

[35] Al-Rasheed NM, Al-Rasheed NM, Hasan IH, Al-Amin MA, Al-Ajmi HN, Mohamad RA (2017). Simvastatin ameliorates diabetic cardiomyopathy by attenuating oxidative stress and inflammation in rats. Oxidative Med Cell Longev.

[36] Frati G, Schirone L, Chimenti I, Yee D, Biondi-Zoccai G, Volpe M (2017). An overview of the inflammatory signalling mechanisms in the myocardium underlying the development of diabetic cardiomyopathy. Cardiovasc Res.

[37] Dinh W, Futh R, Nickl W, Krahn T, Ellinghaus P, Scheffold T (2009). Elevated plasma levels of TNF-alpha and interleukin-6 in patients with diastolic dysfunction and glucose metabolism disorders. Cardiovasc Diabetol..

