# Supplementary material for: Neutrophil: lymphocyte ratio is positively associated with subclinical diabetic cardiomyopathy
Source: BMC Endocr Disord. 2020 Jun 30;20:99. doi: 10.1186/s12902-020-00571-y (PMC7329473; doi:10.1186/s12902-020-00571-y)
Supplement: Supplementary file 1 — Additional file 1 : Supplemental Table 1. Participant characteristics of T2DM with or without cardiac dysfunction. Supplemental Table 2. Echocardiographic features of participants with or without cardiac dysfunction. Supplemental Table 3. ROC analysis for continuous predictor. [file 12902_2020_571_MOESM1_ESM.docx]

Supplemental Table 1. Participant characteristics of T2DM with or without cardiac dysfunction.

| Participant characteristics | T2DM without cardiac dysfunction | T2DM with cardiac dysfunction | *P* value |
| --- | --- | --- | --- |
| N | 462 | 45 |  |
| Sex (male, %) | 293 (63.41%) | 20 (44.44%) | 0.012* |
| Age (years) | 53.43 ± 8.28 | 56.38 ± 8.24 | 0.023* |
| BMI (kg/m^2^) | 23.46 ± 3.54 | 24.75 ± 5.17 | 0.026* |
| WHR | 0.95 ± 0.02 | 0.95 ± 0.01 | 0.980 |
| Duration of diabetes (years) | 6.21 ± 6.02 | 11.56 ± 5.57 | <0.001* |
| FBG (mmol/L) | 9.55 ± 0.13 | 10.93 ± 0.67 | 0.004* |
| 2-hour PBG (mmol/L) | 14.20 ± 4.95 | 15.97 ± 5.16 | 0.023* |
| HbA1c (%) | 9.76 ± 2.46 | 10.35 ± 2.43 | 0.126 |
| ALT (U/L) | 24.36 ± 28.81 | 26.24 ± 24.67 | 0.672 |
| AST (U/L) | 21.77 ± 14.50 | 20.87 ± 31.34 | 0.720 |
| BUN (mmol/L) | 5.25 ± 1.38 | 5.31 ± 1.47 | 0.350 |
| Cr (mmol/L) | 69.86 ± 13.45 | 69.31 ± 15.61 | 0.901 |
| BNP (ng/L) | 47.01± 2.89 | 43.93 ± 6.54 | 0.749 |
| CTNI (ng/mL) | 0.0019 ± 0.0001 | 0.0017 ± 0.0004 | 0.671 |
| CK (U/L) | 76.33 ± 44.06 | 63.38 ± 2.84 | 0.051 |
| CK-MB (U/L) | 1.49 ± 1.12 | 1.65 ± 2.90 | 0.450 |
| Myoglobin (ng/mL) | 19.80 ± 0.66 | 17.00 ± 1.28 | 0.190 |
| TC (mmol/L) | 4.66 ± 1.46 | 5.36 ± 0.62 | 0.002* |
| TG (mmol/L) | 2.19 ± 2.37 | 2.79 ± 0.91 | 0.092 |
| HDL (mmol/L) | 1.08 ± 0.02 | 0.97 ± 0.03 | 0.033* |
| LDL (mmol/L) | 4.05 ± 0.92 | 2.75 ± 0.11 | 0.659 |
| neutrophil | 3.56 ± 0.06 | 3.81 ± 0.20 | 0.229 |
| lymphocyte | 2.01 ± 0.04 | 1.82 ± 0.09 | 0.091 |
| NLR | 1.97 ± 0.05 | 2.31 ± 0.13 | 0.050* |

The data are summarized as the mean ± SD for continuous variables or as a numerical proportion for categorical variables. BMI: body mass index; WHR: waist-to-hip ratio; FBG: fasting blood glucose; 2-hour PBG: 2-hour postprandial blood glucose; HbA1c: hemoglobin A1c; ALT: alanine aminotransferase; AST: aspartate aminotransferase; BNU: urea nitrogen; Cr: creatinine; BNP: type B natriuretic peptide; CTNI: cardiac troponin I; CK: creatine kinase; TC: total cholesterol; TG: triglyceride; HDL: high density lipoprotein; LDL: low density lipoprotein.

Supplemental Table 2. Echocardiographic features of participants with or without cardiac dysfunction.

| Participant characteristics | T2DM without cardiac dysfunction | T2DM with cardiac dysfunction | *P* value |
| --- | --- | --- | --- |
| N | 462 | 45 |  |
| LA diameter | 33.88 ± 3.28 | 34.02 ± 4.20 | 0.789 |
| LVEDD (mm) | 45.84 ± 2.96 | 46.38 ± 3.56 | 0.256 |
| LVESD (mm) | 29.87 ± 1.71 | 30.51 ± 2.32 | 0.021* |
| IVSD (mm) | 8.38 ± 0.71 | 8.93 ± 1.34 | <0.001* |
| EF (%) | 64.44 ± 1.76 | 63.80 ± 2.23 | 0.023* |
| E velocity(cm/s) | 75.94 ± 14.61 | 74.84 ± 14.44 | 0.631 |
| A velocity(cm/s) | 72.03 ± 14.94 | 99.87 ± 15.18 | <0.001* |
| E/A ratio | 1.08 ± 0.21 | 0.76 ± 0.15 | <0.001* |
| e’ velocity(cm/s) | 9.55 ± 0.08 | 6.56 ± 0.17 | <0.001* |
| E/e’ ratio | 8.01 ± 1.00 | 11.44 ± 1.29 | <0.001* |

The data are summarized as the mean ± SD for continuous variables or as a numerical proportion for categorical variables. LA: left atrial; LV: left ventricular; LVEDD: LV diameter in end diastolic; LVESD: LV diameter in end systolic; IVSD: interventricular septal diameter; LVEF: LV ejection fraction; E: the peak early diastolic trans-mitral flow velocity; A: the peak late diastolic trans-mitral flow velocity; e’: the peak early diastolic mitral annular velocity.

Supplemental Table 3. ROC analysis for continuous predictor.

|  | AUC | 95% CI | Specificity | Sensitivity |
| --- | --- | --- | --- | --- |
| Neutrophil | 0.559 | 0.471-0.646 | 0.550 | 0.578 |
| lymphocyte | 0.399 | 0.308-0.489 | 0.115 | 0.670 |
| NLR | 0.865 | 0.818-0.913 | 0.729 | 1.000 |
